# The (cost) effectiveness of an online intervention for pregnant women with affective symptoms: protocol of a randomised controlled trial

**DOI:** 10.1186/1471-2393-14-273

**Published:** 2014-08-14

**Authors:** Hanna M Heller, Annemieke van Straten, Christianne JM de Groot, Adriaan Honig

**Affiliations:** Department of Psychiatry, VU University Medical Center, de Boelelaan 1117, 1081 HV Amsterdam, The Netherlands; Department of Clinical Psychology, Faculty of Psychology and Education & EMGO Institute for Health and Care Research, VU University Amsterdam, Amsterdam, The Netherlands; Department of Obstetrics and Gynecology, VU University Medical Center, de Boelelaan 1117, 1181 HV Amsterdam, The Netherlands; Sint Lucas Andreas hospital, Jan Tooropstraat 164, 1061 AE Amsterdam, Netherlands

**Keywords:** Pregnancy, Affective symptoms, Internet, Perinatal outcome, Postpartum depressive disorder, Self-help intervention, Cost-effectiveness

## Abstract

**Background:**

Women in pregnancy and postpartum have an increased vulnerability to develop an affective disorder. Affective disorders in pregnancy are associated with an increased risk of prematurity, dysmaturity (foetal weight below the 10^th^ percentile as determined by ultrasound) and the development of postpartum depressive disorder. Untreated affective disorders and their complications may also result in considerable costs. Recent meta-analyses showed that interventions during pregnancy are less effective than postpartum interventions probably because of high attrition due to the barriers pregnant women experience with attending sessions outside their homes. An internet-based self-help intervention may overcome these barriers as it can be followed at home, and also in one’s own time. Such internet interventions showed to be effective for decreasing affective symptoms in general.

This randomised clinical trial examines whether an internet-based self-help intervention is effective in the reduction of affective symptoms in pregnancy and postpartum and results in an improvement of the perinatal outcome. We will also determine the cost-effectiveness of the intervention.

**Methods/design:**

We will investigate the effectiveness of a 6 week internet-based self-help problem solving treatment (PST) for affective symptoms in pregnancy. We aim to include 286 women with mild to severe affective symptoms who will be randomly assigned to the internet-based intervention or a waiting list control group. Primary outcome measures are affective symptoms and the perinatal outcome. Secondary outcome measures are quality of life, and economic costs. All assessments are based on self-report and will take place at baseline (T0), 10 weeks later (after completion of the intervention (T1), 4 weeks before the expected day of birth (T2), and 6 weeks after delivery (T3). The control group will be measured at the same moments in time. Analysis will be based on the intention-to-treat principle.

**Discussion:**

If shown (cost) effective, internet-based PST will offer new possibilities to treat pregnant women for affective symptoms, to improve their perinatal outcome and to prevent the development of postpartum depressive disorders.

**Trial registration:**

Nederlands Trial Register: NTR4321

## Background

Women in pregnancy and postpartum have an increased vulnerability to develop a depression or anxiety disorder, both affective disorders. The prevalence of depressive and anxiety disorders during pregnancy is 12 and 11% respectively [1]. The prevalence rate of mild affective symptoms is estimated to be 17% [2]. Depressive and/or anxiety symptoms in pregnancy are often not diagnosed because of overlapping symptomatology with pregnancy itself. They remain therefore often not recognised [3, 4].

Affective disorders are associated with adverse perinatal outcomes such as an increased risk of prematurity, dysmaturity, and decreased breastfeeding initiation [3, 5]. Moreover, antenatal depressive and/or anxiety symptoms are a risk factor for the development of postpartum depressive disorder and therefore for impaired development of the child [6–11]. Therefore, untreated affective disorders and their complications may result in considerable costs [8]. Consequently, prevention of the occurrence of an affective disorder during pregnancy is important for mother, child and society at large.

A recent meta-analysis, which included 28 Randomised Controlled Trials (RCT’s), showed that pre- and postpartum psychological interventions reduced the number of women who developed postpartum depression [9]. The most effective interventions were postpartum (instead of antenatal) individual interventions (instead of group interventions) aimed at women with multiple risk factors (instead of offering it to all women). Attrition was noted as one of the main problems to accomplish a sufficient intervention dose. This is probably due to the fact that pregnant women have many specific barriers to attend sessions outside their home. The following interventions appear to show promise in the reduction of antenatal depressive symptoms and the subsequent prevention of postpartum depressive disorder: lay telephone support, home visits by nurses, and interpersonal therapy (IPT).

An internet-based self-help intervention may overcome pregnancy specific barriers related to face-to-face interventions. Internet interventions are easy accessible, home-based and can be followed in one’s own time. There is no waiting list and there is also a reduction in therapist time and costs. This type of intervention is therefore a promising approach in the treatment of depressive and anxiety symptoms in pregnant women.

In recent years self-help internet programs have become increasingly popular in mental health care. They are based on evidence based psychological treatments. Nowadays, numerous randomised controlled trials are available demonstrating the effectiveness of internet-based self-help interventions for different mental disorders such as depression [12, 13], anxiety [14, 15], alcohol [16], and insomnia [17]. Various treatment modalities have been applied within these trials, for example Cognitive Behavioural Therapy (CBT) and Problem-Solving Therapy (PST). It has been demonstrated that internet self-help treatments provided with support are more effective than those without any support [18]. To our best knowledge no evidence is available on self-help internet interventions for depressive and anxiety symptoms in pregnancy.

We will examine the effectiveness of an intervention based on an existing, evidence based internet self help version of PST [19] compared to a waiting list control condition on (1) the reduction of depressive and anxiety symptoms post intervention, end of pregnancy and 6 weeks postpartum and, (2) the improvement in perinatal outcomes (for example pre-term birth, growth restriction and breastfeeding initiation). We will also (3) determine cost-effectiveness using a societal perspective. We will further explain the intervention in the method section.

## Methods and design

### Study design

**Figure 1:**
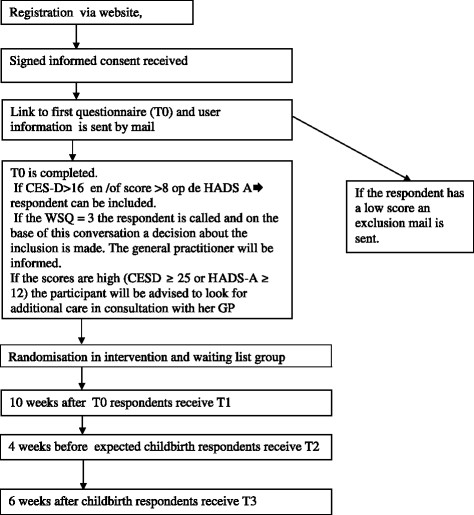
**Flowchart selection, inclusion, intervention and measurement.**

The study is a randomized controlled trial with an active intervention arm and a waiting list control condition. The intervention is based on problem solving treatment and will be offered through the internet. It will be guided by a trained coach. Participants in the control condition are offered access to the same intervention after the last follow-up of the trial (6 weeks postpartum). Both groups are allowed to use concurrent treatment (care-as-usual) as well. This additional care will be monitored.The study protocol, information brochure and informed consent were approved by the Medical Ethics Committee of the VU University Medical Center (registration number 2013.275). Figure 1 displays the flowchart of the study design.

### Inclusion and exclusion criteria

We will include participants who are at risk for developing postpartum depression. More specifically, we will include participants of 18 years and older, who are pregnant but are at least 10 weeks before the expected delivery date, and have at least mild symptoms of depression or anxiety. Symptoms of depression are measured with the Center for Epidemiological Studies Depression scale (CES-D) and symptoms of anxiety with the Hospital Anxiety and Depression Scale- Anxiety subscale (HADS-A) [20, 21]. Patients are eligible if they score at least 16 on the CESD and/or 8 on the HADS-A. Participants who indicate that they either intend to harm themselves or want to attempt suicide (as assessed by one question of the Web Screening Questionnaire (WSQ) [22]) will be excluded. They are advised to consult their general practitioner to obtain more tailored care. Receiving psychiatric treatment including psychotherapy or the use of psychopharmacological agents such as antidepressants is allowed. Any additional treatments will be monitored through patients self-report at the end of study.

### Recruitment

Participants will be recruited from the general Dutch population by means of banners on internet websites and advertisements in newspapers and magazines for pregnant women. These banners and advertisements refer to our study website. The website provides general information about the study and women can register online. All registered women will then be informed in more detail. They will also receive an informed consent form and a link to a baseline questionnaire. Only women who return a signed informed consent, complete the online baseline questionnaire, meet inclusion criteria and do not meet exclusion criteria will be included and randomized. Participants with very severe depressive or anxiety symptoms (CESD ≥ 25 or HADS-A ≥ 12) will be advised to contact their general practitioner as well, since the possibility exists that they need additional treatment. They will not be excluded since there exists evidence that the intervention is also helpful in case of severe depressive symptoms [23, 24].

### Randomisation

Participants will be randomized in a 1:1 ratio. An independent researcher will create a computer generated randomisation scheme, using blocks of 10. This independent researcher will store the randomisation scheme and provide the next randomisation outcome after each inclusion by the researcher. This procedure ensures blind allocation. All participants will then be informed by e-mail on the randomisation outcome.

### Intervention

We adapted the existing, evidence based internet version of PST, for use in pregnant women [25]. We added psychoeducation on pregnancy and affective symptoms and adjusted the example patients in the programs. We named the intervention ‘MamaKits online’. The core assumption of PST is that affective symptoms are generated when people become overwhelmed by practical problems they face in their daily lives. When people are able to make a list of their worries and problems, and learn structured ways to resolve them, they feel less overwhelmed. Then they are better able to cope and this will in turn alleviate their mood [19].

The course takes 6 weeks to complete, with one lesson each week. Each lesson consists of: information, examples and homework assignments. The intervention consists of three steps: (1) participants describe what really matters to them (2) participants write down their current worries and problems and categorize them into three types: (a) unimportant problems (problems unrelated to the things that matter to them), (b) problems which can be solved, and (c) problems which cannot be solved (e.g. the loss of a loved one), (3) participants make a plan for the future in which they describe how they will try to accomplish those things that matter most to them.

The core of the intervention consists of a structured approach to solve potentially solvable problems. It consists of six steps: (1) write a clear definition of the problem, (2) generate multiple solutions to the problem, (3) select the best solution, (4) work out a systematic plan for this solution, (5) carry out the solution, and (6) evaluate as to whether the solution has resolved the problem.

Trained coaches will give weekly feedback on the assignments through e-mail. The coaches will be master level psychology students, psychologists and psychiatrists who will receive a three hour training by an experienced PST therapist and are afterwards closely supervised by the same person. Based on previous experience with e-mail coaching we expect the coaching to take 15–30 minutes per participant per lesson. Feedback is aimed at supporting participants to work through the intervention and assignments, not to develop a therapeutic relationship. The main researcher, an experienced psychiatrist (HH), will check the integrity of the feedback.

### Control group

Participants in the control group are allowed to use ‘treatment as usual’ when needed. The ‘treatment as usual’ will be assessed through patients self-report by means of the questionnaires. Six weeks postpartum they will be offered the intervention.

### Instruments

#### Primary outcomes

**Depressive symptoms** We will use the Dutch version of the CES-D to measure symptoms of depression [20]. This questionnaire has 20 self-rated items, each scored 0–3. The total score range is 0 (no depressive symptoms) to 60 (high number of depressive symptoms). The validity has been tested in different populations. Scores of 16 and higher represent a clinically significant level of depressive symptoms with a sensitivity of 0.82-1.00 and a specificity of 0.69-0.88 [26, 27] and is chosen as an inclusion criterion.

### Suicidality

We will use one question of the WSQ [28] to asses suicidality. We will contact participants with a score 3 by telephone to give them advice about obtaining additional treatment. If needed we will also contact their general practitioner. For that reason we will also ask the telephone number of the participant and the name of het general practitioner.

### Anxiety symptoms

For assessing anxiety symptoms the Dutch version of the HADS A is used. This is a 7 item anxiety subscale of the HADS with item responses on a 0 to 3 scale. Total score range is 0–21. Higher scores indicate more anxiety. The questionnaire is found to be reliable in the paper-pencil version [29] as well as in the internet version [30]. The HADS A has an optimal cut-off ≥ 8 with a sensitivity of 0.89 and a specificity of 0.75 [27].

### Perinatal outcomes

At 6 weeks after delivery we will assess the perinatal complications such as preterm birth (birth of baby less than 37 weeks gestational age), low birth weight, breastfeeding initiation and obstetric complications such as unplanned Caesarean Section, a vacuum extraction or longer duration of hospitalisation. This information will be obtained from participants only.

### Secondary outcomes

#### Depressive symptoms

The Edinburgh Postnatal Depression Scale (EPDS) [31] is a 10 item depression scale developed for women in the postpartum period. Item response varies from 0 to 3. Total score range is 0–30. Cronbach’s alpha was 0.82 in the Dutch paper-pencil version [32]. As the EPDS is not well validated as a screenings instrument in the antepartum period we will not use it as our primary outcome [33]. We will use the EPDS as a secondary measuring instrument for comparison as this scale is widely used in this target population.

### Health care utilization

We will monitor uptake of health care, during the whole study period with the TiC-P [34].We will include all health care utilization of the mother (e.g. visits to general practitioner, to mental health care, to gynaecologist, hospital admissions etc.), as well for the new-born (e.g. additional visits from the midwife or to the child health centre, to the general practitioner and/or the paediatrician) irrespective of the cause or reason for using the service.

### Productivity losses

We will also monitor possible productivity losses. We will register the duration of the maternity leave, frequency and duration of sick leave from work (absenteeism) and the extent a women has worked but less efficient (presenteeism). This will be measured with the SF-HLQ which is incorporated within the TIC-P [34].

### Quality of life

Quality of life is assessed with the Euroqol [34–37]. This is self report questionnaire which measures quality of life and consists of 5 health state dimensions (pain/discomfort, depression/anxiety, mobility, self-care and usual activity) giving an indication of the own health state. The value that the individual or the society may place on a particular health state is expressed in a utility score. This utility score varies by a number between 0 (worst imaginable condition: death) and 1 (perfect health). In the Netherlands the tariff of valuation to determine the utility score is used to calculate quality-adjusted life years (QALYs) by multiplying the utility score with the amount of years the health state exists.

### Demographics

We will assess gender, age, marital status, number and ages of other children, education, profession and ethnicity.

### Assessments

All assessments are based on self-report and will take place online (Table 1). We will measure at baseline (T0), 10 weeks later (although it is possible to finish the intervention in 6 weeks we know that there will be some people who need more time. To ensure that the post-test assessment takes place after completing the intervention we will perform the post-test assessments after 10 weeks.), when the intervention group has finished the intervention (T1), 4 weeks before the expected day of birth (in order to registrate symptoms of relapse (T2), and 6 weeks after delivery (T3). Participants starting the intervention after 24 weeks of pregnancy will not be assessed at T2, since the time period between T1 and T2 will be too small.

**Table 1:** **Assessment time table**

	T0	T1	T2	T3
CES-D	X	X	X	X
HADS	X	X	X	X
WSQ	X			X
EPDS	X	X	X	X
TiC-P	X	X	X	X
Euroqol	X	X	X	X
Demographic variables	X			
Delivery outcome				X

### Sample size and statistical analysis

Analyses will be conducted according to intention-to-treat as well as per protocol principle. To study differences between the conditions we will use linear mixed models with symptoms of depression and anxiety as dependent variables, time indicators and treatment dummy as predictors, since these types of models account for drop out over time. We will compute both within group effect sizes and between group effect sizes. The within group effect sizes are standardized differences of (predicted) means of the follow up scores and baseline scores. The between group effect sizes concern differences between within group effect sizes. We assume that the between group effect size (Cohen’s d) at the end of the study will be at least 0.40, because this has been demonstrated in a meta-analysis on supported internet treatments in general [13] and in previous studies using the same problem solving internet treatment [19, 25]. Using an alpha of 0.05 (two-tailed), a statistical power (1-bèta) of 0.80, and an attrition rate of 30% [19] we need 143 respondents in each arm. All analyses will be conducted using SPSS for Windows, version 20 and STATA 10.0.

### Economic analysis

The economic analyses are undertaken from a societal perspective, taking into account intervention costs, direct medical costs, direct non-medical costs and indirect costs during the study period [38]. Indirect costs refer to lost resources and opportunities resulting from the disease. We will assess health service uptake and production losses at T0, T1, T2 and T3 based on the TIC-P. Full economic costs due to care utilisation and production losses are obtained from the national manual for cost prices in the health care sector. The intervention costs concern the costs related to the internet application, the advertisement and the training/salary of the coaches. Indirect costs include production losses both in paid labour and in the domestic sphere. In addition, both costs due to work loss and work cut-back are assessed, as work cut-back in patients with affective symptoms may be substantial. To calculate indirect costs the friction cost method is used, thereby taking the replacement of sick employees into account, resulting in more conservative cost estimates than the human capital approach. As the timeframe used in the present study is relatively short, costs are not discounted nor corrected for inflation.

To account for the possible non-normality of the cost data, sample errors and 95% confidence intervals are based on resampling methods (bootstrapping) using 2,500 replications.

A cost-effectiveness analysis assesses the costs per recovered patient [39]. Quality of life is assessed at each assessment point with the Euroqol, and the average quality of life during the study period is calculated.

A cost-utility analysis is similar to a cost effective study, but assesses the incremental costs per QALY gained during the study period (instead of the incremental costs per recovery).

We will report the incremental cost-effectiveness ratio. The Incremental cost-effectiveness ratio (ICER) is the ratio between the difference in costs (between experimental and control condition) and the difference in effects (between experimental and control condition), where costs is the average annual per capita cost and effects is the percentage of participants that recovered from their depression.

We will report the ICER, the scatter of the bootstrapped ICERs on the ICER plane and the ICER acceptability curve for the probability that the intervention dominates care as usual for a series of willingness-to-pay ceilings. Use will be made of the pertinent guidelines for health economic evaluation.

## Discussion

The aim of this study is to investigate the effectiveness of an internet-based self-help intervention on depressive and anxiety symptoms in pregnancy. Furthermore we investigate the effects on pregnancy outcome. Through a calculation of costs and quality of life gains an indication can be given on the cost effectiveness of the intervention. This study has several limitations.

First not all women have access to the internet. This might be especially true for those with increased vulnerability for affective symptomatology during pregnancy such as social disadvantaged women in particular those with illiteracy, low educational level and low income. However especially in Western countries internet is broadly available and therefore this intervention is widely accessible during pregnancy.

Second, drop out rate may be high in the non intervention group due to lack of motivation. In order to counter this, therapy is offered at a later stage, 6 weeks post delivery.

A third limitation is the use of self rating instruments only. However since this intervention is aimed at affective symptoms and not at affective disorders a formal diagnosis seems not necessary.

The strength of the study is that we examine a short practical intervention for a specific group with mild to severe affective symptoms, which has proven to be (cost) effective in other populations. The broad inclusion and the use of the internet will allow us to create a high external validity. Furthermore the study is not only aimed at depressive, but also at anxiety symptoms, which is a common, but often neglected symptom in pregnancy and postpartum.

In case effectiveness is proven we will be able to offer an effective treatment of affective symptoms in pregnancy and their complications, reach many women and making therapy easier accessible.

## Abbreviations

PST: Problem solving treatment
RCT’s: Randomised Controlled Trials
IPT: Interpersonal therapy
CBT: Cognitive Behavioural Therapy
CES-D: Center for Epidemiologic Studies Depression scale
HADS: The Hospital Anxiety and Depression scale
WSQ: Web Screening Questionnaire
EPDS: Edinburgh Postnatal Depression Scale
TiC-P: Trimbos/iMTA questionnaire for Costs associated with Psychiatric Illness
SF-HLQ: Short Form- Health and Labour Questionnaire
ICER: Incremental cost-effectiveness ratio
QALYs: Quality-adjusted life years.
